# The clinicopathological and prognostic significances of IGF-1R and Livin expression in patients with colorectal cancer

**DOI:** 10.1186/s12885-022-09961-y

**Published:** 2022-08-05

**Authors:** Zhenling Zhang, Yuxin Zhang, Si Lao, Jian Qiu, Ziang Pan, Xiaoying Feng

**Affiliations:** 1 Department of Gastroenterology, the Second Hospital of Dalian Medical University, Dalian, 116023 China; 2 Department of Pathology, the Second Hospital of Dalian Medical University, Dalian, 116023 China

**Keywords:** Insulin-like growth factor 1 receptor (IGF-1R), Livin, Colorectal cancer, Tumorigenesis, The Cancer genome atlas (TCGA)

## Abstract

**Background:**

Colorectal cancer (CRC) is the third most common cancer worldwide. However, limited effective biomarkers are associated with the tumorigenesis and prognosis of CRC.

**Methods:**

The present study identified potential signatures from The Cancer Genome Atlas (TCGA) database and further validated the identified biomarkers in CRC tissues by immunohistochemistry (IHC).

**Results:**

The expression of insulin-like growth factor 1 receptor (*IGF-1R*) and *Livin* gene was significantly upregulated in CRC samples compared to the adjacent normal samples in the TCGA dataset. IHC indicated that IGF-1R and Livin protein levels are increased in CRC and adenoma tissues compared to normal tissues. Notably, the IGF-1R protein levels differed significantly between adenoma and CRC. The elevated IGF-1R and Livin expression was associated with CRC clinicopathological features, including age, gender, histological subtype, individual cancer stages, nodal metastasis, and TP53-mutant in TCGA. Additionally, the IGF-1R promoter methylation level was closely related to CRC. Consistent with the TCGA study, IHC indicated that overexpressed IGF-1R and Livin proteins were independent risk factors for stage and metastasis. A marked correlation was established between IGF-1R and Livin expression in CRC, while the survival map showed no significant correlation with CRC. Kaplan–Meier survival curves showed that CRC patients with high IGF-1R or Livin expression had a prolonged overall disease-free survival than those with low expression in TCGA.

**Conclusion:**

IGF-1R and Livin are associated with CRC tumorigenesis and might be valuable for novel biomarker identification and targeted therapeutic strategy development.

**Supplementary Information:**

The online version contains supplementary material available at 10.1186/s12885-022-09961-y.

## Introduction

Colorectal cancer (CRC) is the third most common malignant tumor and the second most common cause of death worldwide. According to the GLOBOCAN project of the World Health Organization (WHO) Cancer Research Center, the number of new CRC cases in 2020 is about 1.93 million worldwide, and the number of deaths is about 940,000 [1]. By 2030, the global burden of CRC will increase by 60%: the number of new cases will exceed 2.2 million, and the number of deaths will exceed 1.1 million [2]. The incidence of CRC varies globally, with colon cancer being the highest rates in European regions, Australia/New Zealand, and Northern America and with rectal cancer being the highest rates in Eastern Asia, while CRC incidence tends to be low in most regions of Africa and in South Central Asia [1]. Age-standardized incidence and mortality rates for CRC in the United States of America has decreased significantly recently, but increased in China [3]. Several risk factors, including age, hereditary components, chronic intestinal inflammation, obesity, excessive alcohol and red meat consumption, smoking, and lack of physical exercise, have been identified for CRC [4–6]. Despite the advancement in comprehensive therapy, the long-term survival of CRC patients remains unsatisfactory [7, 8]. The poor therapeutic outcome of patients with CRC is mainly due to local recurrence and distant metastasis [9]. Therefore, understanding the pathogenesis of CRC and finding effective therapeutic targets is imperative*.*

Insulin-like growth factor 1 receptor (IGF-1R) belongs to the tyrosine kinase receptor family. The elevated expression and activity of IGF-1R are observed in many cancer types and are associated with tumor cell proliferation, survival, anti-apoptosis, and drug resistance [10–12]. IGF-1R triggers various intracellular signaling cascades in colonic mucosal cells, enhancing cell cycle progression and inhibiting apoptosis [13]. IGF-1R inhibition results in the G1 cell cycle arrest and a significant decrease in CRC cell proliferation, survival, and radioresistance [14, 15]. A previous study demonstrated that IGF-1R is overexpressed in human colorectal cancer HT-29 cell lines. Cyclolignan picropodophyllin (PPP, an IGF-1R inhibitor) inhibits the proliferation and migration of HT-29 cell lines in a dose-dependent manner and decreases the expression/phosphorylation of IGF-1R, extracellular signal-regulated kinase 1/2 (ERK1/2), and Akt [16]. The overexpression of the inhibitor of apoptosis protein (IAP) family, including baculoviral IAP repeat-containing protein-7 (BIRC7), facilitates apoptosis evasion in CRC [17]. BIRC7 is also referred to as Livin or melanoma inhibitor of apoptosis. *BIRC7/Livin* also interacts with the second mitochondria-derived activator of caspases through the baculoviral IAP repeat domain to promote the caspase activation in the cytochrome c pathway [18]. *BIRC7/Livin* potentiates migration and invasion of CRC cells partially via nuclear factor-kappa B (NF-κB)-mediated epithelial-mesenchymal transition [19]. Although both IGF-1R and Livin are potential biomarkers for CRC, only a few studies have investigated their role in normal mucosa-colorectal adenoma-colorectal cancer progression.

The analysis of differential gene expression between diverse cancer types and adjacent normal tissues across The Cancer Genome Atlas (TCGA) cohort revealed higher *IGF-1R* and *Livin* gene expression in patients with colon adenocarcinoma (COAD) compared to the adjacent normal tissues from the Sangerbox, GEPIA2, and UALCAN portal datasets. The current study also used TCGA-COAD to explore the clinical significance, patient survival, and correlation between IGF-1R and Livin expression and CRC. In agreement with the TCGA study, CRC (*n* = 60), colorectal adenoma (*n* = 30), and adjacent normal tissues (*n* = 10) and their clinicopathological data were selected, and the expression and distribution of IGF-1R and Livin proteins in various intestinal mucosal tissues was detected, and the correlation between IGF-1R and Livin expression and clinicopathological parameters, occurrence, development, and clinical prognosis of CRC patients was analyzed. These data may reveal a novel oncogenic function and the clinical value of IGF-1R and Livin in CRC.

## Materials and methods

### Patients and surgical specimens

A total of 60 patients with primary CRC who underwent surgical resection at the General Surgery Department of the Second Affiliated Hospital of Dalian Medical University (Dalian, China) comprised the CRC group. The cohort was in Dukes’ stages A, B, C, and D (*n* = 15 cases). None of the patients had received chemotherapy, radiotherapy, or other anti-cancer treatment before the operation. Biopsies from 10 cases of adjacent non-tumor tissues comprised the control group. Thirty patients who underwent colonoscopy and endoscopic polypectomy, endoscopic mucosal resection (EMR), or endoscopic submucosal dissection (ESD) at the Department of Gastroenterology of the Second Affiliated Hospital of Dalian Medical University were regarded as the colorectal adenoma group, including 8 cases of tubular adenoma and 22 cases of villous tubular adenoma. All tissue specimens were confirmed pathologically, and all the clinicopathological data were complete. Samples from patients with diabetes, acromegaly, dwarfism, cachexia, severe organ failure, or other malignant tumors were excluded. This research protocol has been reviewed by the Ethics Committee of the Second Affiliated Hospital of Dalian Medical University and agreed that the project would be carried out on the premise of protecting the rights and interests of subjects (approval No. 132, ethical review 2021, the Second Affiliated Hospital of Dalian Medical University).

### Data processing and analysis

For gene expression, clinical significance, and survival analysis, the TCGA-COAD datasets from Sangerbox (http://sangerbox.com/), GEPIA2 (http://gepia2.cancer-pku.cn/), and UALCAN portal (http://ualcan.path.uab.edu/) were used. The significant difference was estimated by Student’s t-test considering unequal variance for gene expression and clinical significance analysis. The normalized average counts from the adjacent normal tissue samples served as the threshold to filter the samples. Then, only samples with counts above the threshold were used for survival analysis. Survival plots were drawn by comparing the top 30% (high-expression group) and the bottom 30% filtered samples (low-expression group). Survival curves of overall survival (OS) and disease-free survival (DFS) were estimated by Kaplan–Meier analysis. The *P*-values were calculated by log-rank test.

The correlation analysis was performed with the GEPIA2 dataset in the “colorectal adenocarcinoma” and “adjacent normal tissues” from the TCGA cohort by Spearman’s chi-square test analysis, using the non-log scale for calculation and the log-scale axis for visualization.

### Immunohistochemistry (IHC)

The specimens were fixed in formalin and embedded with paraffin before slicing into 3-μm-thick sections. After deparaffinization using xylene and dehydration using graded ethanol, the sections were immersed into citrate buffer at pH 7.2–7.4 for antigen retrieval. Then, the sections were probed with IGF-1R (1:200; Abcam Cat# ab39522, RRID:AB_2122268) and Livin (1:200; Proteintech Cat# 27543–1-AP, RRID:AB_2880901) antibodies at 4 °C overnight and 37 °C for 1 h, followed by phosphate-buffered saline (PBS) washes. Subsequently, the sections were incubated with a biotinylated secondary antibody (Zhongshan Golden Bridge Biotech Co. Ltd., Beijing, China) at 37 °C for 30 min. The immunoreaction was developed by incubation with diaminobenzidine (DAB) for 10 min. Hematoxylin was used for counterstaining and alcohol gradient for dehydration. Finally, the sections were sealed with neutral gum and observed under a light microscope (Olympus Bx-51, Japan). All the slides were separately examined and scored by two experienced pathologists blinded to the study design.

Staining results: IGF-1R and Livin were observed as yellow or brownish-yellow staining in the cell membrane. Five random fields were examined at high magnification for each sample. The staining intensity (no staining is 0, slight staining is 1 point, moderate staining is 2 points, and deep staining is 3 points) and ratios of positive cells (0–25% refers to 0 points, 26–50% is 1 point, 51–75% is 2 points, > 76% is 3 points) were determined using the couple score and semiquantitative method. The final results were multiplied by two scores mentioned above: 0–1 point, negative (−); 2–4 points, weak positive (+); 5–7 points, positive (++); 8–9 points, strong positive (+++).

### Statistical analysis

The data were analyzed using SPSS 20.0 statistical software (IBM Co., Armonk, NY, USA). Mann–Whitney U test or Kruskal–Wallis test was used for enumeration data, and Spearman’s chi-squared test was used for correlation analysis. The test standard α = 0.05 and *P* < 0.05 indicated a statistically significant difference.

## Results

### IGF-1R is frequently overexpressed in primary CRC tumors

**Fig. 1:**
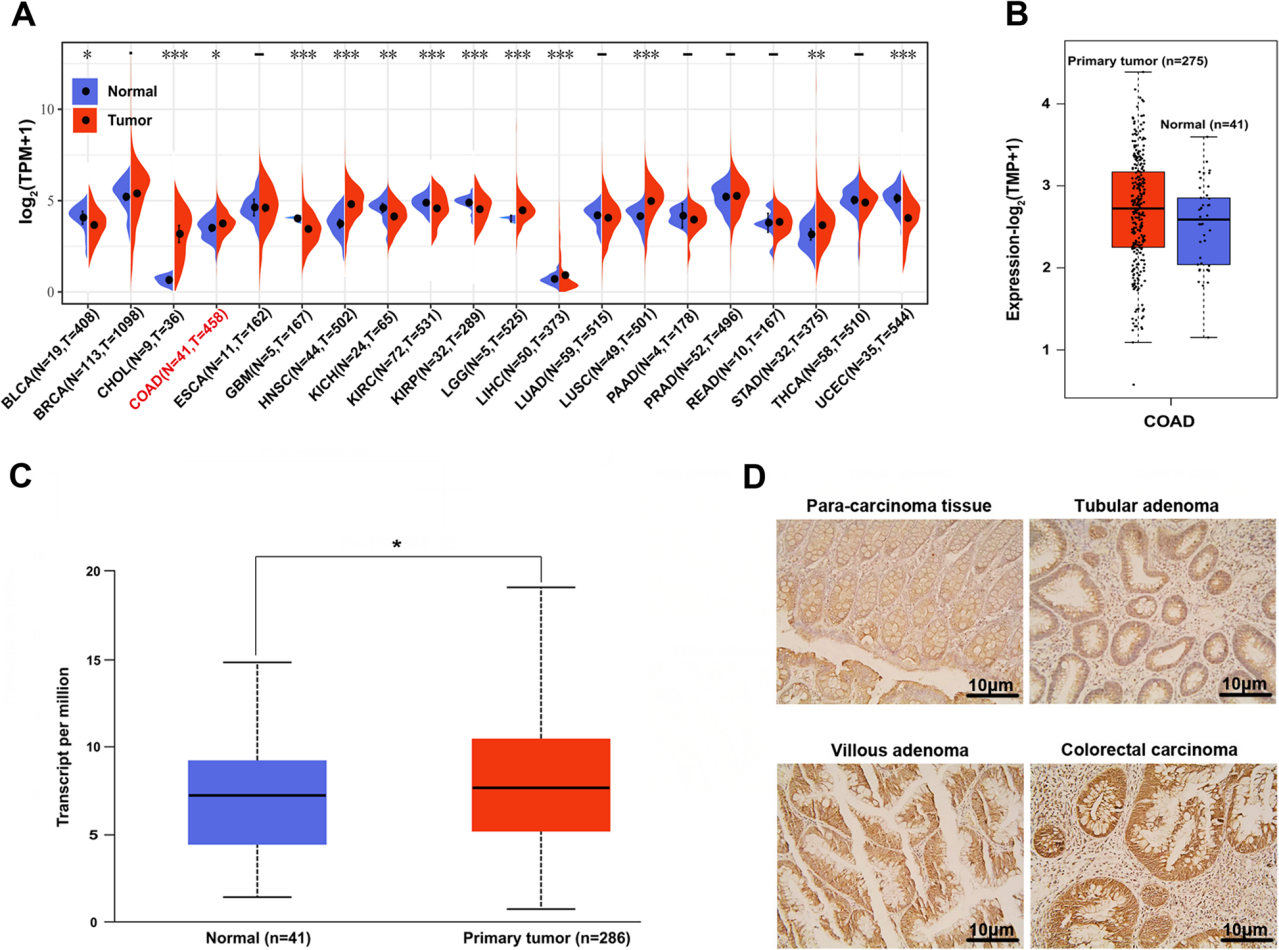
IGF-1R expression in CRC patients. *IGF-1R* gene expression between diverse cancer types (**A**) and primary COAD (**B** and **C**) from TCGA database. **D** IGF-1R protein expression in paracancerous, tubular adenoma, villous adenoma, and CRC patients tissues by IHC staining (400×, scale = 10 μm). IGF-1R, insulin-like growth factor 1 receptor; COAD, colorectal adenocarcinoma; TCGA, The Cancer Genome Atlas; CRC, colorectal cancer; IHC, immunohistochemistry. *P < 0.05, **P < 0.01, and ***P < 0.001

The analysis of the *IGF-1R* gene expression between diverse cancer types and adjacent normal tissues across The Cancer Genome Atlas (TCGA) cohort revealed high gene expression in COAD (*n* = 458) compared to the adjacent normal tissues (*n* = 41) (*P* < 0.05, Fig. 1A) from Sangerbox dataset. Similarly, upregulated *IGF-1R* gene expression was also observed in primary COAD in GEPIA2 dataset (*n* = 275, Fig. 1B) and UALCAN portal dataset (*n* = 286, *P* < 0.05, Fig. 1C). Furthermore, the IHC results showed significantly higher levels of IGF-1R in tubular adenoma, villous adenoma, and CRC tissues compared to the adjacent normal tissues, as assessed by the Mann–Whitney U test, and the difference between adenoma and CRCs was statistically significant (Fig. 1D, Table 1). These results demonstrated that IGF-1R is commonly overexpressed in CRC and may play a critical role in precancerous polyps growth and malignant transformation.

**Table 1:** Expression of IGF-1R and Livin in CRC, colorectal adenoma and adjacent normal tissues

Groups	IGF-1R expression				Total	Livin expression				Total
	–	+	++	+++		–	+	++	+++	
Adjacent normal tissues	8 (80%)	2 (20%)	0	0	10	10 (100%)	0	0	0	10
Colorectal adenoma	5 (16.7%)	13 (43.3%)	9 (30%)	3 (10%)	30	9 (30%)	16 (53.3%)	4 (13.3%)	1 (3.33%)	30
Colorectal carcinoma	9 (15%)	17 (28.3%)	18 (30%)	16 (26.7%)	60	23 (38.3%)	21 (35%)	13 (21.7%)	3 (10%)	60
Total	22	32	27	19	100	42	37	17	4	100

### Livin expression is upregulated in CRC

**Fig. 2:**
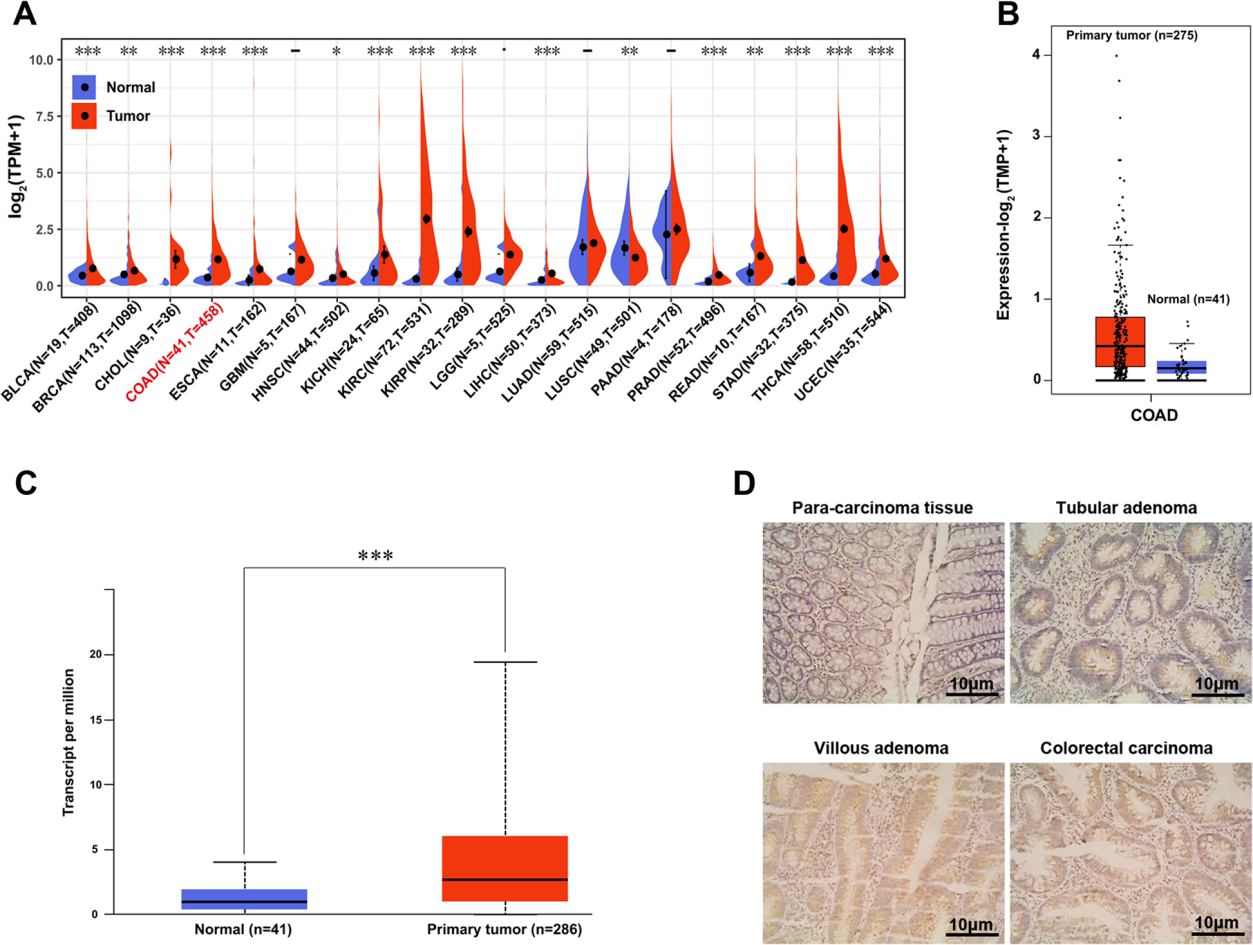
Livin expression in CRC patients. *Livin* gene expression between diverse cancer types (**A**) and primary COAD (**B** and **C**) from TCGA database. **D** Livin protein expression in paracancerous, tubular adenoma, villous adenoma, and CRC patients tissues by IHC staining (400×, scale = 10 μm). COAD, colorectal adenocarcinoma; TCGA, The Cancer Genome Atlas; CRC, colorectal cancer; IHC, immunohistochemistry. *P < 0.05, **P < 0.01, and ***P < 0.001

Next, the Livin (also known as BIRC7) expression in primary CRC tissues was analyzed from TCGA cohort. The gene expression was significantly upregulated in primary COAD compared to the adjacent normal tissues in the Sangerbox dataset (*P* < 0.001, Fig. 2A). Specifically, the upregulation of *Livin* gene expression in COAD was validated in two datasets (GEPIA2 and UALCAN) of paired tumor and adjacent normal samples from the TCGA study (Fig. 2B and C). Livin IHC was conducted in a cohort of 100 patients with localized CRCs, adenoma, and adjacent normal tissues to further analyze Livin expression and validate these findings. The staining showed that the level of Livin protein in the colorectal adenoma and cancer groups was significantly higher than that of the adjacent normal tissues; however, no significant difference was detected between the colorectal adenoma and cancer groups (Fig. 2D, Table 1). These results demonstrated that the high level of Livin protein is related to the occurrence of CRC, and it may not be a crucial factor in the progression of adenoma to CRC.

### Overexpression of IGF-1R is an independent predictor of oncogenic function in CRC patients

**Fig. 3:**
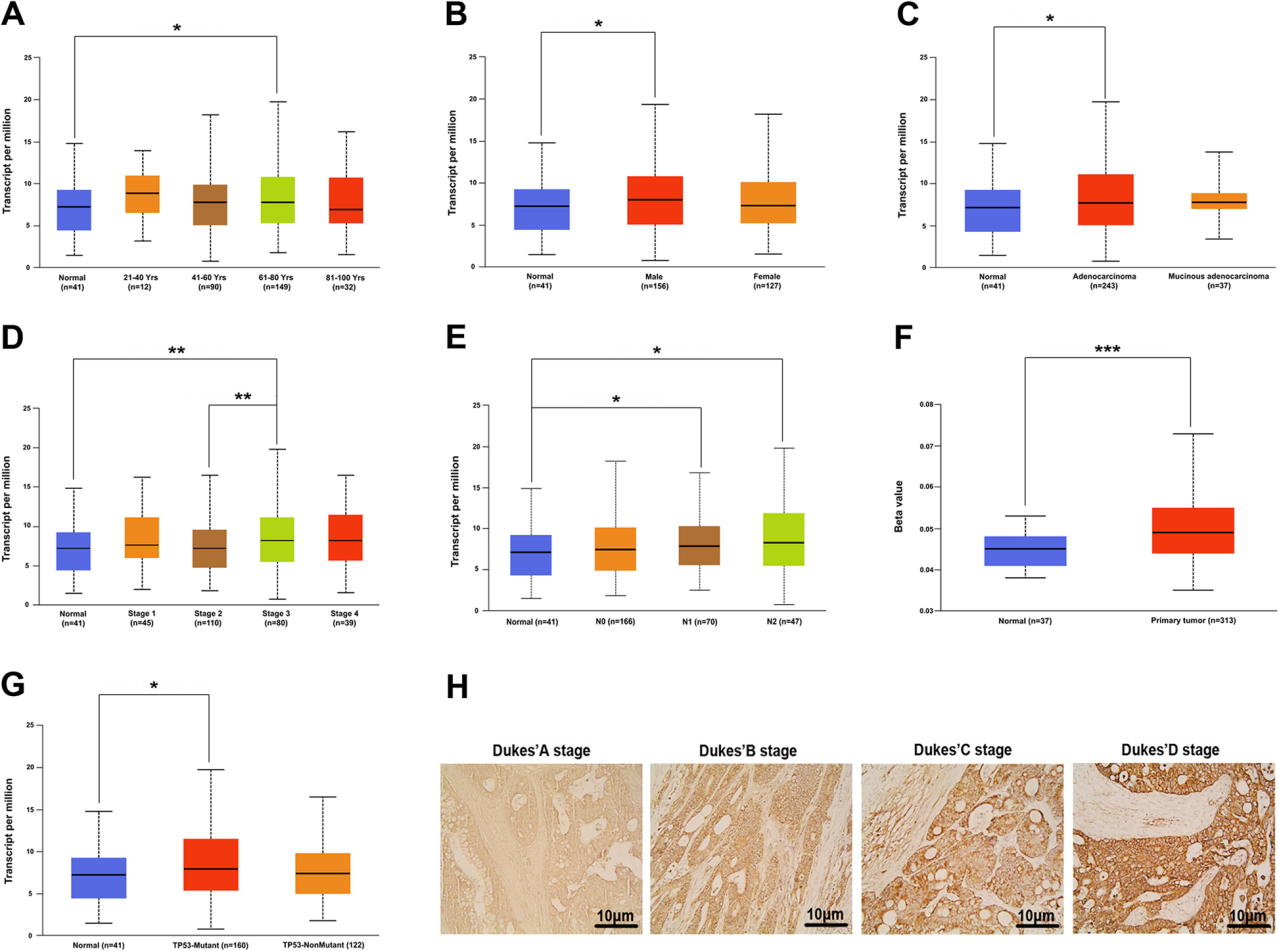
Elevated IGF-1R expression is associated with the tumorigenesis and progression of CRC. The correlation between *IGF-1R* gene expression and CRC clinicopathological features, including age (**A**), gender (**B**), histological subtype (**C**), individual cancer stages (**D**), nodal metastasis status (**E**), promoter methylation (**F**), and TP53-mutant (**G**) in TCGA. **H** The expression of IGF-1R protein in Dukes’ A, B, C, and D of CRC (400×, scale = 10 μm). IGF-1R, insulin-like growth factor 1 receptor; CRC, colorectal cancer; TCGA, The Cancer Genome Atlas. *P < 0.05, **P < 0.01, and ***P < 0.001

Next, the clinicopathological significance of the IGF-1R gene and protein expression was evaluated in CRC patients. A significant correlation was established between *IGF-1R* gene expression and clinicopathologic features, including age (61–80 years), gender (male), histological subtype (adenocarcinoma), individual cancer stages (stage 3), nodal metastasis status (N1 and N2), and TP53-mutant (Fig. 3A-E and G). In addition, the promoter methylation level of IGF-1R was closely related to COAD (Fig. 3F). Also, the correlation between IGF-1R protein expression and clinicopathological features in CRC was evaluated. Consistent with the TCGA study, IGF-1R protein overexpression was associated with the depth of invasion, Dukes’ stage, lymph node metastasis, and distant metastasis in patients with CRC (all *P* < 0.05, Table 2), and with the increase in Dukes’ stage, the expression level of IGF-1R protein increased significantly (Fig. 3H, Table 2). However, no correlation was established between IGF-1R protein expression and age, gender, diameter, location, histological subtype, and differentiation (Table 2). These results demonstrated that IGF-1R might be used as a biomarker to evaluate the condition of CRC patients.

**Table 2:** The clinicopathologic significance of IGF-1R and Livin expression in patients with CRC (*N* = 60)

Characteristics	Number	IGF-1R expression				*P*-value	Livin expression				*P*-value
		–	+	++	+++		–	+	++	+++	
Age,years						0.824					0.760
< 50	8	1	2	4	1		4	2	2	0	
50–70	37	6	10	8	13		11	15	8	3	
≥ 71	15	2	5	6	2		8	4	3	0	
Gender						0.847					0.889
Male	33	6	7	11	9		13	11	7	2	
Female	27	3	10	7	7		10	10	6	1	
Tumor diameter, cm						0.852					0.831
< 5	40	6	11	12	11		17	12	8	3	
≥ 5	20	3	6	6	5		6	9	5	0	
Tumor location						0.059					0.918
Right colon	16	2	5	6	3		4	9	2	1	
Left colon	18	1	6	7	4		5	7	6	0	
Rectum	26	6	6	5	9		14	5	5	2	
Pathological type						0.363					0.847
Adenocarcinoma	56	8	15	18	15		22	19	12	3	
Mucinous adenocarcinoma	4	1	2	0	1		1	2	1	0	
Histological type						0.598					0.328
Moderately-Well differentiated	54	8	15	16	15		20	18	13	3	
Poorly differentiated	6	1	2	2	1		3	3	0	0	
Depth of invasion						0.004					0.291
Tis-T1	8	2	5	1	0		4	3	1	0	
T2-T4	52	7	12	17	16		19	18	12	3	
Dukes’ stage						0.000					0.044
A	15	6	6	2	1		9	5	1	0	
B	15	2	7	5	1		5	7	3	0	
C	15	1	3	6	5		6	4	5	0	
D	15	0	1	5	9		3	5	4	3	
Lymph node metastasis						0.000					0.022
Absent	33	8	13	8	4		16	12	4	1	
Present	27	1	4	10	12		7	9	9	2	
Distant metastasis						0.000					0.032
Absent	45	9	16	13	7		20	16	9	0	
Present	15	0	1	5	9		3	5	4	3	

### Clinical application value of Livin expression in CRC

**Fig. 4:**
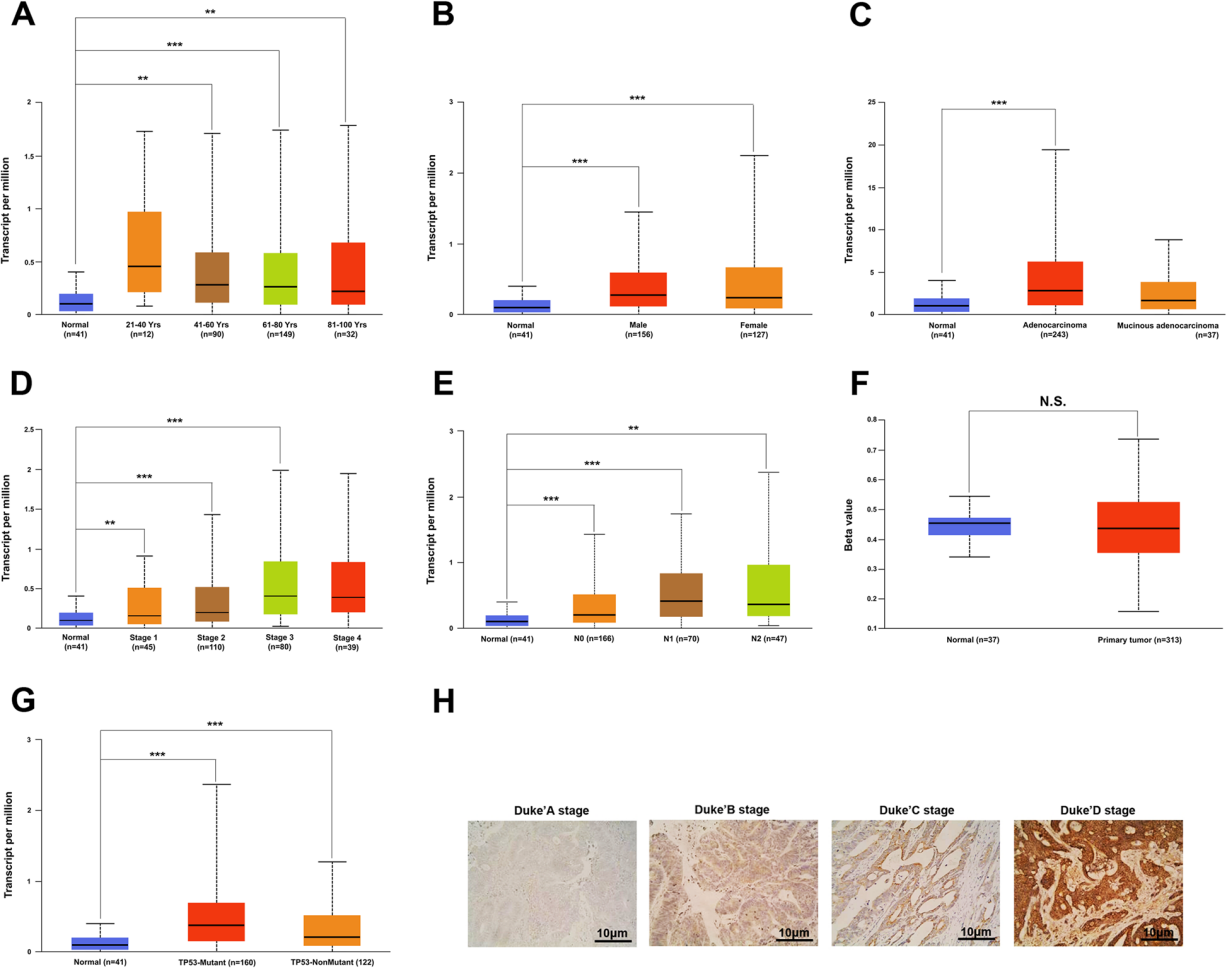
Elevated Livin expression is associated with the tumorigenesis of CRC. The correlation between *Livin* gene expression and CRC clinicopathological features, including age (**A**), gender (**B**), histological subtype (**C**), individual cancer stages (**D**), nodal metastasis status (**E**), promoter methylation (**F**), and TP53-mutant (**G**) in TCGA. (**H**) The expression of Livin protein in Dukes’ A, B, C, and D of CRC (400×, scale = 10 μm). CRC, colorectal cancer; TCGA, The Cancer Genome Atlas. **P < 0.01, ***P < 0.001, and N.S. no significance

The association between Livin gene and protein expression and the clinicopathological features of CRC was analyzed. As shown in Fig. 4A–G, a significant correlation was established between *Livin* gene expression and clinicopathological features, including age (41–60, 61–80, and 81–100 years), gender (male and female), histological subtype (adenocarcinoma), individual cancer stages (1, 2, and 3), nodal metastasis status (N0, N1, and N2), and TP53 mutation status (TP53-mutant and TP53-non-mutant), while no correlation was established in the promoter methylation level of Livin. Moreover, Spearson’s chi-square test analysis showed that Livin protein overexpression was an independent risk factor for stage and metastasis but was not associated with age, gender, tumor size, primary tumor location, pathological type, histological type, or depth of invasion (Fig. 4H, Table 2).

### IGF-1R and Livin are not prognostic markers of CRC

**Fig. 5:**
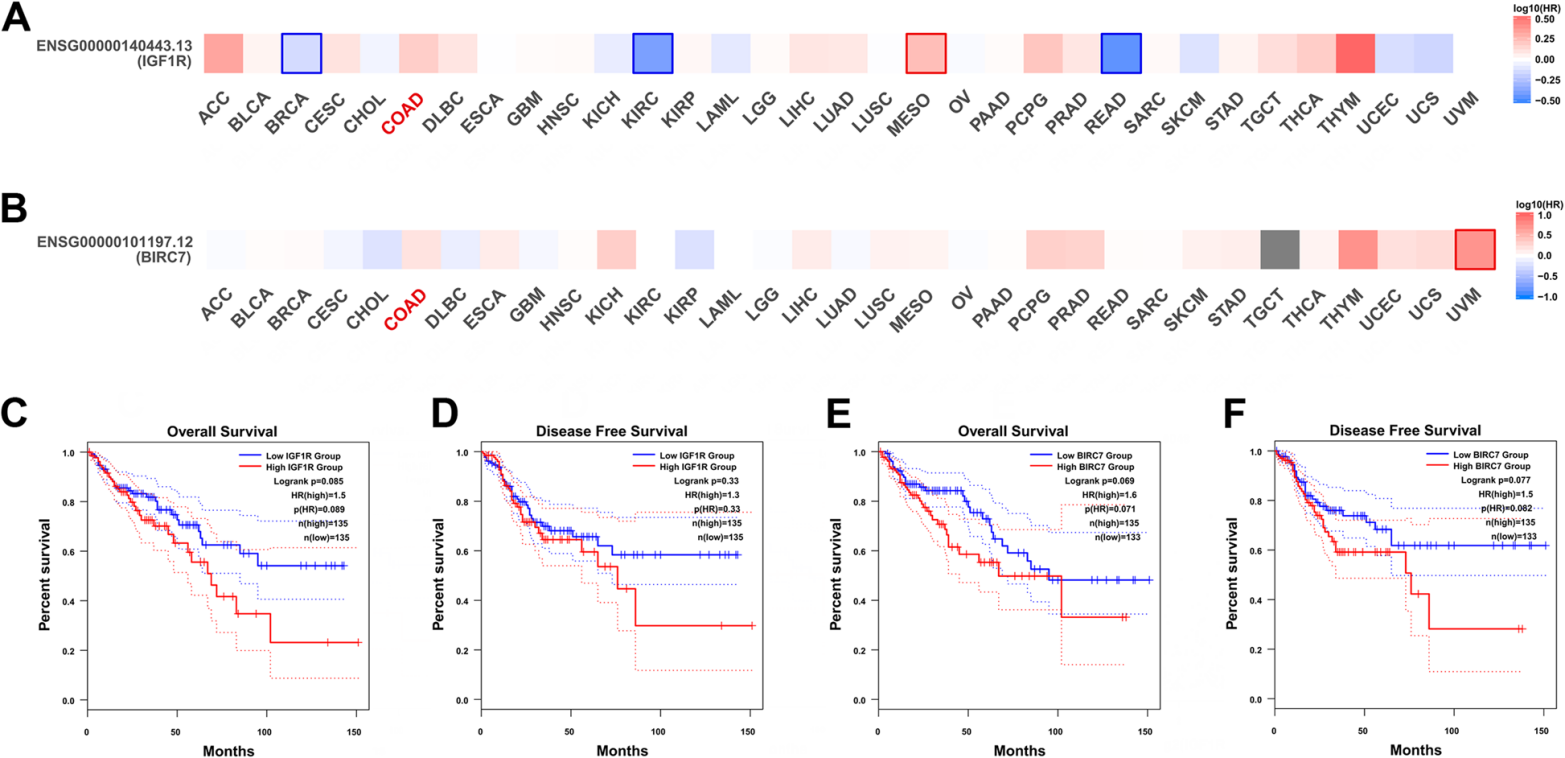
Elevated IGF-1R and Livin expression are not associated with poor prognosis in CRC patients. **A** and **B** Survival map analyses of *IGF-1R* and *Livin* gene expression in TCGA. **C** and **D** Kaplan–Meier survival curves of OS and DFS for CRC patients between high- and low-IGF-1R expression groups in TCGA. **E** and **F** Kaplan–Meier survival curves of OS and DFS for patients with CRC between high and low Livin expression groups in TCGA. The blue curve represents the low IGF-1R and Livin expression groups. The red curve represents the high IGF-1R and Livin expression group. IGF-1R, insulin-like growth factor 1 receptor; TCGA, The Cancer Genome Atlas; OS, overall survival; DFS, disease-free survival; CRC, colorectal cancer

**Fig. 6:**
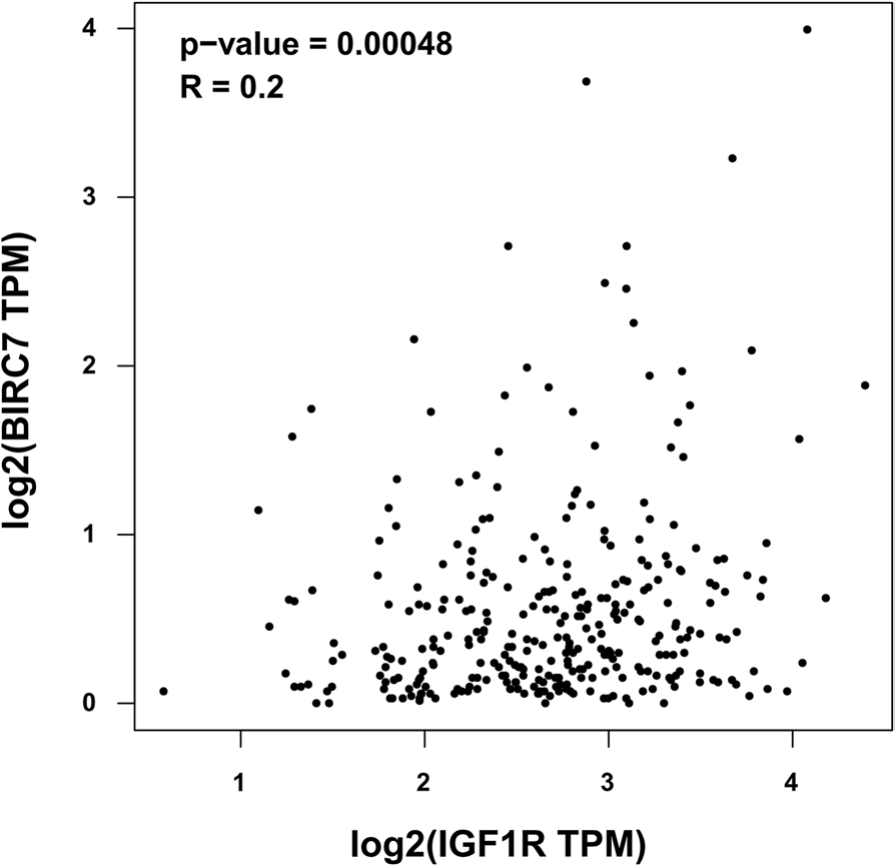
Correlation between IGF-1R and Livin expression in CRC. The correlation between IGF-1R and Livin expression in TCGA-COAD by Spearman’s chi-square test analysis. The non-log scale for calculation and the log-scale axis for visualization. TCGA, The Cancer Genome Atlas; COAD, colorectal adenocarcinoma

The correlation between IGF-1R and Livin expression and patients’ OS and DFS was estimated by Kaplan–Meier analysis. The survival map showed that both IGF-1R and Livin are not significantly correlated with COAD from the TCGA dataset (Fig. 5A and B). the patient cases from TCGA-COAD with survival data were sorted into the high- and low-expression of IGF-1R and Livin groups. As shown in the Kaplan–Meier survival curves, CRC patients with high IGF-1R or Livin expression showed a longer OS and DFS than those with low expression based on the log-rank test (*P* > 0.05 for both OS and DFS, Fig. 5C-F). Moreover, Spearman’s chi-squared test analysis established a marked correlation between IGF-1R and Livin expression in COAD (*R* = 0.2, *P* = 0.00048, Fig. 6).

## Discussion

The present study demonstrated that *IGF-1R* and *Livin* genes are highly expressed in CRC and the other cancer types, such as cholangiocarcinoma and kidney, liver, and lung cancers, through the TCGA database [20–23]. Also, the expression of *IGF-1R* and *Livin* genes was increased in the 275 CRC cases from the GEPIA2 dataset and 286 colorectal cancer cases from the UALCAN portal dataset. IGF-1R may play a role in the neoplastic progression of CRC because it is related to both cellular proliferation and differentiation [24]. However, low-level IGF-1R expression is found in some invasive cancers, which promotes cancer cell dedifferentiation to a mesenchymal type morphology with loss of cell adhesion [25]. The expression of Livin is associated with tumor development and progression in CRC by increasing tumor cell motility and inhibiting apoptosis [26]. The occurrence and development of CRC is a multi-stage complex process, with the dynamic evolution of normal epithelial, proliferative polyp, adenoma, cancer, and metastasis. The results of IHC staining showed a significant correlation between the expression of IGF-1R protein and neoplastic progression from normal mucosa to adenomatous polyps and finally to CRC. The high expression of Livin protein is related to the occurrence of CRC, but it may not be a critical factor in the progression of adenoma to CRC.

Furthermore, the clinical importance of IGF-1R and Livin expression in CRC patients was investigated at the mRNA and protein levels in two independent cohorts and found that both molecules were overexpressed. Expression of IGF-1R increases with tumor size in CRC [27]. *Livin* mRNA expression is strongly related to CRC invasive depth but not to clinical tumor stage, differentiation, lymph node metastasis, tumor morphological category and pathological type, and patient’s age and gender [28]. According to the TCGA study, the levels of IGF-1R and Livin were associated significantly with age, gender, histological subtype, individual cancer stages, nodal metastasis status, and TP53 mutation status in CRC relative to the adjacent normal tissues. *TP53* is a well-known tumor suppressor gene that encodes p53 and is frequently inactivated by mutation or deletion in most human CRCs [29, 30]. In addition, the promoter methylation level of IGF-1R, but not Livin, was closely related to CRC. Moreover, our results showed that high levels of IGF-1R and Livin were significantly correlated with stage and metastasis. However, no correlation was established between IGF-1R and Livin protein expression and other clinicopathological features, such as age, gender, diameter, location, differentiation, and histological subtype from sixty patients with CRC. Our results are inconsistent with those reported in the TCGA database as well as other studies, possibly due to regional and population differences, as well as insufficient sample size and methodology in this study.

Several studies have shown that CRC patients with negative expressions of IGF-1R and Livin had significantly higher accumulative survival rate and longer mean survival duration than those with positive expression of IGF-1R and Livin [18, 26, 31]. Multivariate analysis of potential prognostic factors showed that IGF-1R expression and worsened performance status are independent predictors of poor outcomes [32]. Additionally, high expression of Livin may influence the prognosis of CRC [33]. Both survival map and Kaplan–Meier survival curves showed that high expression levels of IGF-1R and Livin were not correlated significantly with the short OS of patients with CRC. The clinical outcome of CRC varies greatly depending on the aggressiveness of individual tumors. Many patients experience disease recurrence following radical surgery. Nonetheless, the TCGA dataset demonstrated that neither IGF-1R nor Livin serves as an independent prognostic marker for CRC patients, which was opposite to the previous findings. Thus, additional prognostic biomarkers may accurately assess the risk to guide personalized chemotherapy. These results showed a marked correlation between IGF-1R and Livin expression in COAD by Spearman’s chi-square test. In future studies, confocal fluorescence staining would be employed to study the co-localization of IGF-1R and Livin proteins in CRC tissues to determine whether double-positive expression promotes the occurrence and development of CRC more than single-positive expression.

## Conclusions

In summary, IGF-1R and Livin oncogenes are highly expressed in CRC patients. Both can be considered biomarkers for the stage, metastasis, and risk of carcinogenesis. IGF-1R may drive neoplastic initiation and progression along the colorectal normal mucosa-polyp-cancer sequence. In addition, these findings indicated that IGF-1R and Livin are not independent prognostic markers for patients with CRC. Nonetheless, further investigations are needed to substantiate the current findings.

## Supplementary Information

**Additional file 1: Fig. S1.** The negative and positive control expression of IGF-1R and Livin proteins. (40×, scale = 20 μm).

## Data Availability

The datasets analyzed during the current study are available from The Cancer Genome Atlas (TCGA) database, the web links are Sangerbox (http://sangerbox.com/), GEPIA2 (http://gepia2.cancer-pku.cn/), and UALCAN portal (http://ualcan.path.uab.edu/). Along with the above links, IGF-1R accession number: 147370 and Livin accession number: 605737. Other datasets used and/or analyzed during the current study are available from the corresponding author on reasonable request.
