# Supplementary figures and images for: The clinicopathological and prognostic significances of IGF-1R and Livin expression in patients with colorectal cancer
Source: BMC Cancer. 2022 Aug 5;22:855. doi: 10.1186/s12885-022-09961-y (PMC9354317; doi:10.1186/s12885-022-09961-y)

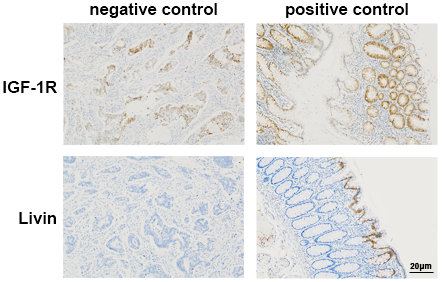


**Fig. S1. The negative and positive control expression of IGF-1R and Livin proteins.** (40×, scale = 20 μm)

Supplement: Supplementary file 1 — Additional file 1: Fig. S1. The negative and positive control expression of IGF-1R and Livin proteins. (40×, scale = 20 μm). [file 12885_2022_9961_MOESM1_ESM.docx]
